# Malnutrition and Erythropoietin Resistance among Patients with End-Stage Kidney Disease: Where Is the Perpetrator of Disaster?

**DOI:** 10.3390/nu14245318

**Published:** 2022-12-14

**Authors:** Wiktoria Feret, Krzysztof Safranow, Ewa Kwiatkowska, Aleksandra Daniel, Kazimierz Ciechanowski

**Affiliations:** 1 Clinical Department of Nephrology, Transplantology and Internal Medicine, Pomeranian Medical University, 70-001 Szczecin, Poland; 2 Department of Biochemistry and Medical Chemistry, Pomeranian Medical University, 70-001 Szczecin, Poland; 3 Internal Medicine Student Science Association, Clinical Department of Nephrology, Transplantology and Internal Medicine, Pomeranian Medical University, 70-001 Szczecin, Poland

**Keywords:** malnutrition, dialysis, inflammation, cytokines, adipokines, erythropoietin resistance, obesity

## Abstract

Background: Hemodialyzed patients with poor erythropoietin response tend to have low volume of visceral adipose tissue and score high on malnutrition-inflammation score. This study investigates in-depth the role of leptin and chosen cytokines in the development of malnutrition-inflammation syndrome (MIS) and erythropoietin resistance. Methods: Eighty-one hemodialyzed patients with erythropoietin-treated anemia were enrolled in the study. Their body composition was measured. Erythropoietin resistance index was calculated. Blood samples for leptin, IL-6, IL-18, TNF-alpha, and IL-1-alpha serum levels were drawn. Results: Leptin showed negative correlation with erythropoietin resistance index (ERI), whilst IL-6 showed the opposite. IL-6 seemed to be linked more to HD parameters and vintage, while TNF-alpha and leptin were more dependent on body composition. IL-18 and IL-1-alpha did not affect nutritional parameters nor ERI. Conclusion: Modulation of adipokine- and cytokine-related signaling is a promising target in tempering malnutrition in hemodialyzed, and thus achieving better outcomes in anemia treatment. Large clinical studies that target the inflammatory response in hemodialysis, especially regarding IL-6, TNF-alpha, and leptin, would be of great worth.

## 1. Introduction

Malnutrition is a significant and growing problem among patients treated with hemodialysis, affecting 23–60% of individuals [1,2,3]. It is linked not only with increased mortality, but also with substantial decrease in quality of life as well [4,5]. Despite technological development giving clinicians better tools to assess nutritional status, such as professional body composition analyzers, proper instruments and interventions to impede the burden of undernourishment in this population are still not well established. A fatal combination of inflammation, hypercatabolism, altered appetite with insufficient food intake, and dialysis-associated factors is thought to play a crucial role in development of malnutrition and its complications [6]. Protein-energy wasting (PEW) relates to the situation in which an individual progressively loses his fat stores, muscle mass and albumin levels in the course of chronic kidney disease (CKD). Malnutrition-inflammation syndrome (MIS) is another, wider expression used to describe nutritional alternations seen in PEW, but with additional impact on oxidative stress and uremic toxins upregulating the inflammatory cascade [7]. MIS is directly linked with erythropoietin hyporesponsiveness in hemodialyzed individuals and makes treatment of anemia, the most common complication of end-stage renal disease (ESRD), very challenging [8,9]. Chronic inflammation is associated with higher levels of hepcidin, the master regulator of iron metabolism in humans. Hepcidin prevents iron release from the macrophages by degrading ferroportin and contributes to anemia development independently from other factors, such as lower endogenous EPO production in the kidneys [10,11]. We believe that erythropoietin resistance index (ERI) can be used as an additional record while screening for malnutrition. In one of our previous studies, we found that hemodialyzed individuals who responded better to EPO not only scored lower in Kalantar-Zadeh’s malnutrition-inflammation score [12], which was mostly consistent with previous data, but also presented with notably higher overall fat mass and visceral fat volume, while muscle mass did not affect ERI [13]. This is why authors hypothesized that adipose tissue in the first place is crucial in preventing EPO hyporesponsiveness and decided to further investigate that. Adipose tissue is known to be hormonally active, releasing adipocytokines, such as leptin, adiponectin, and resistin, which together control energy expenditure and satiety. Nevertheless, its function goes far beyond that and is still being investigated in many fields. It is thought to play role in immune response and erythropoiesis as well [14,15,16,17]. In this single-center cohort study, authors aimed to investigate which of the chosen inflammatory markers (leptin, IL-18, IL-6, IL-1α, TNFα) may contribute to malnutrition and its consequences the most, and further reflected upon possible interventions, based on the most up-to-date literature. 

## 2. Materials and Methods

This study obtained approval of the Bioethical Committee of Pomeranian Medical University in Szczecin (KB-0012/88/03/19).

**Figure 1:**
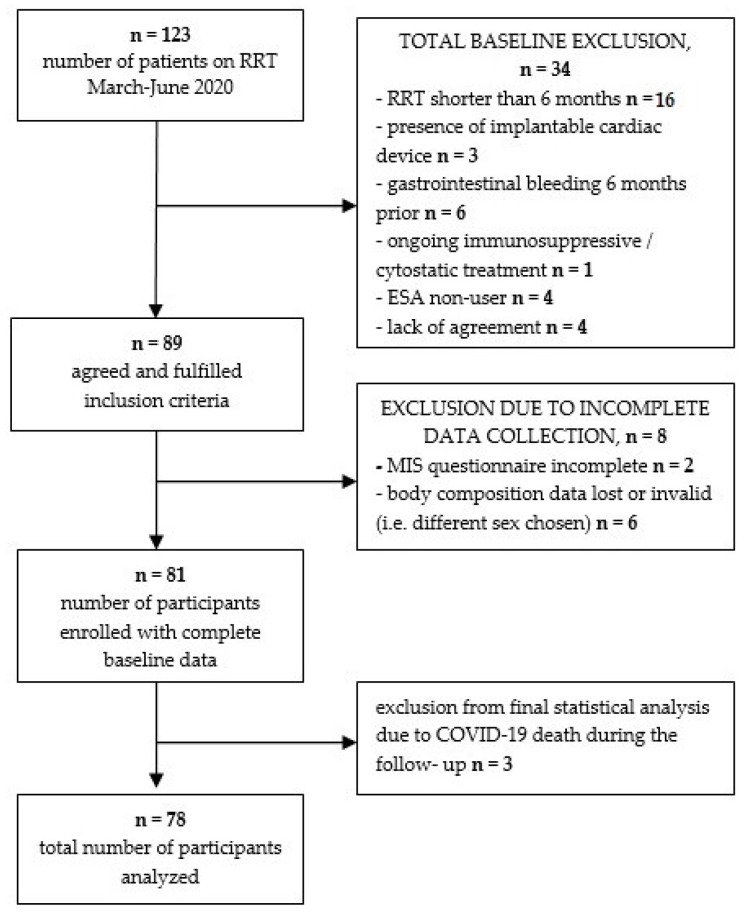
Study group recruitment.

The study focused on patients undergoing renal replacement therapy via hemodialysis in Nephrology Department of The Independent Public Hospital No. 2 in Szczecin, Poland. Time of enrollment was March–June 2020. Detailed group recruitment with baseline and further exclusion criteria is shown below (Figure 1).

Nutritional assessment was performed two-way. First, body composition analysis of each individual was performed using a professional Medical Body Composition Analyzer by SECA (Seca mBCA 525) following the producer’s manual. BCA was made after dialysis session. Seca mBCA 525 uses 8-point bioimpedance to measure body composition parameters. Before each analysis, height, weight and waist circumference had to be entered manually to derive results such as: body mass index (BMI) (kg/m^2^), fat free mass (FFM) (%), fat free mass index (FFMI) (kg/m^2^), fat mass (FM) (%), fat mass index (FMI) (kg/m^2^), total body water (TBW) (%), phase angle (φ) (°), and visceral adipose tissue (VAT) (liters). Taking all these into account, the analyzer assigned each individual into one of four body composition groups: increasing sarcopenic obesity, increasing thinness, increasing muscle mass, and increasing obesity. Then, every individual was scored with a MIS questionnaire [16] which consisted of four parts: (1) patient’s related medical history of last 3–6 months (change in end-dialysis dry weight, dietary intake, gastrointestinal symptoms, functional capacity and co-morbidity including years on dialysis), (2) physical examination regarding signs of fat and muscle loss performed by a qualified physician, (3) body mass index, and (4) laboratory parameters (albumin and transferrin). Total MIS score was calculated at the end, with the maximum possible score being 30 points (http://www.touchcalc.com/calculators/mis (accessed on 7 March 2020)). 

Blood samples to determine levels of leptin, TNF-alpha, IL-6, IL-1-alpha, and IL-18 were collected during the mid-week hemodialysis session, in addition to routine monthly biochemical workup performed in our Dialysis Center. Each blood sample was centrifuged, divided into two or more (if possible) Eppendorf tubes and frozen at −70 °C. One tube was used for leptin levels ELISA measurement, using a kit from Euroimmun Poland. The second tube was for measuring TNF-alpha, IL-6, IL-1-alpha, and IL-18 levels, using Luminex kits by Biotechne. 

The erythropoietin resistance index (ERI) was determined as an average weekly erythropoietin dose/kg body weight/average hemoglobin (g/dL), over the last 6 months.

Mortality was assessed after 18 months of follow-up. The initial number of participants included was 81. Due to SARS-CoV2 spread during the time of the 18-month follow-up, three of the patients enrolled died. The authors excluded them from the final analyses, as little was known about the disease at that time. As it affected mortality in a sudden manner, the authors wanted to preserve the “natural”, previously observed mortality pattern in our group of hemodialyzed patients.

### Statistical Analysis

Statistical analysis was performed using Statistica 13 software (StatSoft, Tulsa, OK, USA). The Shapiro–Wilk test was used to check whether the distributions of quantitative variables were significantly different from normal (*p* < 0.05). We used non-parametric Mann–Whitney U-test to compare groups. Correlations were studied by means of Spearman’s rank correlation coefficient (ρ). Data were described as mean ± SD or median (interquartile range—IQR). *p*-values were considered significant when <0.05. To find independent determinants of leptin levels, we additionally performed multivariate analysis using the general linear model (GLM) with log-transformed leptin concentration as the dependent variable. Standardized Beta coefficient and its 95% confidence interval (95%CI) was presented for each independent variable. Similar multivariate analysis was performed for log-transformed ERI as the dependent variable. To assess survival, we calculated the total number of patients who died, and among those we extracted individuals who died due to cardiovascular events. Groups of survivors and deceased during the 18-month follow up were compared in terms of body composition and laboratory findings. Power analysis was not performed in the study group.

## 3. Results

Seventy-eight individuals with complete data were included in the study. All of them were Caucasian, 47 were male (60.3%). Only male/female gender categories were taken into account. Detailed group characteristics can be found in Table 1.

### 3.1. Leptin

In our study, we found statistically significant correlations between circulating leptin levels and the following parameters (Table 2):

In this study group, leptin levels did not correlate in any way with age (ρ = 0.13, *p* = 0.258), phase angle (ρ = 0.23, *p* = 0.06), dialysis vintage (ρ = −0.023, *p* = 0.81), TNF-alpha (ρ = 0.02, *p* = 0.85), IL-6 (ρ = −0.06, *p* = 0.62), IL-1-alpha (ρ = −0.006, *p* = 0.95), IL-18 (ρ = −0.11, *p* = 0.33), hepcidin (ρ = 0.06, *p* = 0.61), eGFR (ρ = 0.03, *p* = 0.79), HD sessions per week (ρ = −0.08, *p* = 0.48), albumin (ρ = 0.17, *p* = 0.15), transferrin (ρ = 0.19, *p* = 0.10), nor ferritin levels (ρ = −0.003, *p* = 0.98) (data not shown in Table 2).

Due to the multitude of statistically significant correlations, we decided to additionally use general linear model to look for independent determinants of leptin level. Leptin level was independently associated with sex (higher in women), uric acid levels, percentage of muscle mass, visceral adipose tissue, and BMI (Table 3).

Authors compared leptin levels between groups of different ERI. Median ERI in our group was 4.89. Individuals with ERI values lower than median had significantly higher leptin levels than those whose ERI ranged above median (Table 4, *p* = 0.017).

According to body composition analyses, authors compared leptin levels in each subgroup. There was no statistically significant difference of leptin levels between “sarcopenic obesity” and “obesity”; “thinness” and “muscle mass” subgroups also were not different. We found differences between:

- “sarcopenic obesity” vs. “thinness”, *p* = 0.029 (median: 19.63 ng/mL vs. 5.71 ng/mL)
- “obesity” vs. “muscle mass”, *p* = 0.0001 (median: 56.99 ng/mL vs. 8.08 ng/mL)
- “sarcopenic obesity” vs. “muscle mass”, *p* = 0.026 (median: 19.63 ng/mL vs. 8.08 ng/mL)
- “obesity” vs. “thinness”, *p* = 0.0008 (median: 56.99 ng/mL vs. 5.71 ng/mL)

Median ERI in each subgroup was as follows: sarcopenic obesity—2.8, IQR = 4.215; obesity—2.9, IQR =6.69; thinness—6.01, IQR =8.03; muscle mass—6.5, IQR =7.155.

### 3.2. TNF-Alpha

In our study, we found statistically significant correlations of TNF-alpha levels and following parameters (Table 5):

In this study group, TNF-alpha levels were not associated with MIS total score (ρ = 0.21, *p* = 0.063), BMI (ρ = −0.06, *p* = 0.072), VAT (ρ = 0.04, *p* = 0.054), TBW (ρ = −0.12, *p* = 0.24), muscle% (ρ = −0.16, *p* = 0.068), phase angle (ρ = −0.21, *p* = 0.11), ERI (ρ = 0.08, *p* = 0.51), dialysis vintage (ρ = 0.02, *p* = 0.61), eGFR (ρ = −0.19, *p* = 0.29), HD sessions per week (ρ = −0.01, *p* = 0.89), IL-6 (ρ = 0.16, *p* = 0.22), IL-18 (ρ = 0.01, *p* = 0.81), leptin (ρ = 0.02, *p* = 0.81), hepcidin (ρ = 0.19, *p* = 0.71), albumin (ρ = 0.004, *p* = 0.43), transferrin (ρ = −0.07, *p* = 0.32) nor ferritin levels (ρ = 0.10, *p* = 0.38) (data not shown in Table 5).

TNF-alpha levels were significantly higher in deceased of any cause during 18-month follow up than in survivor group, *p* = 0.019 (median 4.8 pg/mL vs. 4.4 pg/mL). TNF-alpha levels did not differ between groups of survivors and deceased due to cardiovascular reasons.

While comparing TNF-alpha levels among groups of different body composition, the only difference was seen between “increasing sarcopenic obesity” and “increasing muscle mass” subgroups (median 4.15 pg/mL vs. 3.53 pg/mL; *p* = 0.033).

### 3.3. IL-6

In our study, we found statistically significant correlations between IL-6 levels and the following parameters (Table 6): 

IL-6 level did not correlate with any other body composition parameter besides phase angle. It was not associated with MIS total score (ρ = 0.22, *p* = 0.11), IL-18 (ρ = 0.19, *p* = 0.21), TNF-alpha (ρ = 0.16, *p* = 0.22), leptin (ρ = −0.06, *p* = 0.62), albumin (ρ = −0.15, *p* = 0.32), ferritin (ρ = 0.05, *p* = 0.23), transferrin (ρ = −0.13, *p* = 0.18), hepcidin (ρ = 0.04, *p* = 0.06), nor eGFR (ρ = −0.08, *p* = 0.91). Comparing subgroups in terms of body composition, we did not find a significant difference in IL-6 levels between any of them.

IL-6 was shown to be an independent determinant of ERI value in the general linear model, *p* = 0.0069 (Table 7).

### 3.4. IL-1α and IL-18

In our study group, IL-1α levels did correlate with TNFα (rho = 0.233, *p*= 0.043) and IL-6 (rho = 0.358, *p*= 0.002) levels. IL-1α had no association with ERI (ρ = 0.12, *p* = 0.27), total MIS score (ρ = 0.08, *p* = 0.43), any of the body composition compounds, age (ρ = −0.13, *p* = 0.98), or dialysis-related data, such as dialysis vintage (ρ = 0.22, *p* = 0.47) or HD sessions per week (ρ = −0.14, *p* = 0.66). Levels of this cytokine did not differ significantly between body composition subgroups.

IL-18 did not show any significant correlation with any of the aforementioned parameters at all in our database.

### 3.5. Association of Studied Cytokines and Body Composition with ERI and MIS Score—Summary

Summary of correlations of studied cytokines with ERI and MIS score can be found below (Table 8 and Table 9).

## 4. Discussion

Inflammation is thought to play a crucial role in the development and progression of numerous diseases, with chronic kidney disease being no exclusion [18]. In patients undergoing hemodialysis, several anomalies can be observed. Due to strict dietary requirements in ESRD and often a lack of clinical dietician support, patients fail to maintain a proper nutritional status. Their fluid balance is hard to control, as well as calorie and protein intake which might be insufficient. Avoiding certain foods, together with impaired appetite in uremic state, makes them prone to develop protein-energy wasting (PEW). The majority of hemodialyzed patients develop a syndrome called MIS (malnutrition-inflammation syndrome) or MIA (malnutrition-inflammation-atherosclerosis). The occurrence of MIS/MIA is known to reduce their quality of life and is linked to greater mortality in this population [19,20]. Inflammation in hemodialyzed may be triggered by uremic toxins [21,22], type of HD access (fistula vs. central venous catheter) [23] as well as underlying conditions. Chronic inflammation can directly affect erythropoiesis and erythropoietin responsiveness, as well as deteriorate level of nutrition [24,25]. In one of our previous studies, we found that erythropoietin resistance index (ERI) is strongly dependent on BMI, fat mass, visceral fat volume, total body water and phase angle, as well as total MIS score and IL-6 levels [13]. That is why authors decided to investigate in-depth how a wider panel of cytokines of choice: IL-6, IL-18, IL-1alpha, TNFalpha, and leptin, which is an adipokine excreted in adipose tissue, influence erythropoietin resistance and nutritional parameters in the population of hemodialyzed patients. 

Based on our results, we can clearly see that the only cytokines that affected ERI were IL-6 and leptin. Levels of circulating IL-6 were positively correlated with high ERI, meaning poor erythropoietin response. Leptin levels, on the other hand, showed negative correlation with ERI value, which can indicate a possible protective function of leptin in this case. Moreover, what’s worth noticing is that in our group IL-6 levels did not show correlation to MIS total score nor any of the basic body composition parameters, besides phase angle, which is an indicator of cell wall stability [26]. This stands in contrast to some of the previous data, in which IL-6 levels were positively correlated with MIS score and total body water and negatively correlated with fat tissue index and lean tissue index [27]. A two-year observational study by Beberashvili et al. showed that fat mass and phase angle correlated with IL-6 levels at baseline: every 1-pg/mL increase in IL-6 was associated with reductions in fat mass and phase angle, but changes in IL-6 serum levels over two years did not significantly correlate with changes in body composition [28].

In our study, higher concentrations of IL-6 were observed in individuals whose dialysis vintage was longer and who had more HD sessions per week. Thus, we assume that IL-6 is a cytokine which is mostly linked to RRT duration per se and does not relate to body composition parameters a lot. In available literature, a time-dependent increase in IL-6 serum levels of HD patients was also seen [28,29,30]. 

IL-6 promotes expression of hepcidin, a ferroportin-degrading molecule, and thus iron release is impeded, preventing proper hematopoiesis. In our opinion, IL-6 serum level reduction is a promising target in overcoming erythropoietin resistance. There are some interesting emerging data regarding this topic. A recent randomized controlled trial of ziltivekimab (NCT02868229), a novel anti-IL-6-ligand antibody, showed that it can significantly reduce EPO requirements in ESRD patients [31]. Another IL-6 inhibitor, tocilizumab, was used to treat inflammation-induced anemia in cancer patients with good clinical effect [32]. Other group of research on reducing IL-6 levels in hemodialyzed is based on choosing adequate dialysis technique. Donati et al. found that asymmetric cellulose acetate (ATA) dialyzers were superior when it comes to removing IL-6 over polymethylmethacrylate (PMMA) dialyzers [33]. A randomized controlled trial by Weiner et al. aimed to examine the efficacy of medium cut-off dialyzers vs. standard high-flux dialyzers in removal of uremic toxins during four- and 24-week period. The MCOD group demonstrated significantly larger reduction ratios for complement factor D, free k light chains, TNF-alpha, and b2-microglobulin (*p* < 0.001 for all), but not for IL-6 [34]. These data support the need for further research into IL-6 signaling blockade as it has potential to improve hematopoiesis. More effective means of blood purification have yet to be implemented to reduce IL-6 levels in hemodialyzed patients. Further research regarding such interventions and their influence on inflammation-driven malnutrition are certainly needed.

As we previously mentioned, in our study group, leptin was negatively correlated with ERI. This adipokine also showed a significant negative correlation with MIS total score, fat-free mass, percentage of muscle mass and total body water. Positive correlations of leptin levels were seen with body weight, BMI, BSA, fat mass, fat mass index, and visceral adipose tissue. Sex, muscle mass, visceral adipose tissue, uric acid levels, and BMI were shown to be independent determinants of leptin levels in our study group (Table 2). Comparing groups in terms of body composition chart placement, leptin levels, and ERI, it can be noted that individuals that were grouped as obese or sarcopenic obese had higher leptin levels and lower ERI than those considered thin or muscular. No correlation with any of the examined cytokines was seen. These data suggest that high leptin levels are generally seen in individuals who are well-nourished and score low on MIS scale. Women tend to have higher leptin levels, which is consistent with available data and is thought to be caused by leptin–estrogen interplay [35,36]. Leptin is an interesting adipokine with a multitude of biological actions. It not only takes part in regulating appetite and energy expenditure, but is also linked to erythropoiesis and bone-mineral metabolism [37,38]. Similar to albumin, it is a reverse-acute phase protein in the population undergoing HD [39]. In a study by Risovic et al., leptin turned out to be associated with two independent determinants of mortality in malnourished and overhydrated patients on HD, and was significantly lower in individuals who died during 12-month follow-up. In this study, patients with low leptin levels also had low BMI, high TBW, low fat tissue index, and low fat free mass index [40,41], which stands in line with our results. In a small study by Rafieian-Kopaei, higher leptin levels were associated with higher hemoglobin and lower EPO requirements in ESRD [39]. Abi et al. studied the association between leptin levels and resting energy expenditure (REE) in a CKD stage 3–5 KDIGO population without hemodialysis and found a positive correlation of leptin and REE independently of sex-energy expenditure was greater with higher leptin levels [36]. Authors of the aforementioned study hypothesized that leptin antagonist or LepR-blocker administration in this population may help ameliorate PEW and cachexia in the course of CKD, just as it did in animal studies [42,43]. In the population of hemodialyzed patients, an interesting phenomenon of inverse epidemiology seems to relate to leptin levels as well: higher leptin means better nutrition. We suppose it might be due to central or peripheral leptin resistance, which in this case seems to play a protective role against malnutrition and appetite decrease. According to Axelsson, both visceral fat mass and leptin can be a predictor of EPO response in hemodialyzed [44]. Leptin stimulates erythropoiesis by activating bone marrow and spleen HSC niches [38]. Sturzebecher et al. administered leptin to mice with lipodystrophy. In lipodystrophy, due to the inability to store fat in adipose tissue, it is stored in the liver and consequently, causes vascular abnormalities similar to those seen in obesity. In this study, leptin reduced endothelial inflammation by inhibiting endothelial to mesenchymal transformation and reduced vascular leakage [45]. Thus, we hypothesize that it might be one of the protective mechanisms of leptin in our population, although of course animal models cannot be directly addressed to humans. It is possible that either leptin-signaling inhibitors or supraphysiological doses of leptin, together with increased energy intake and/or intradialytic parenteral nutrition [46], could be a strategy to ameliorate MIS and erythropoietin resistance in malnourished HD individuals, but this hypothesis surely needs further studies.

TNF-alpha is another important biomarker of inflammation, often described together with IL-6 as a red flag of uremic milieu. It was primarily thought to reduce appetite, promote muscle protein breakdown and endothelial dysfunction and its elevated levels were linked with age, visceral obesity and overhydration in ESRD [47]. It is still poorly understood whether high TNF-alpha is one of the reasons or rather one of the effects of uremia. Novel data on TNF-alpha and malnutrition are lacking, but in a recent study by Caldiroli et al. higher levels of TNF-alpha were seen in patients with higher MIS scores in univariate analysis [48]. Zhong et al. assessed the level of circulating mitochondrial DNA (MtDNA) and correlated it with TNFalpha and IL-6 levels in maintenance HD patients. Not only were TNFalpha levels higher in ESRD patients comparing to healthy individuals, but it correlated positively with MtDNA copies. MtDNA is released during cell death, so high levels of TNF-alpha can be somehow linked to impaired cell wall stability and necrosis [49]. In our study, we observed a positive correlation of TNF-alpha and age, IL-1alpha, as well as fat mass and fat mass index. It was negatively correlated with fat-free mass. It did not correlate with ERI or MIS score in any way. Additionally, TNF-alpha levels were higher in individuals who died during 18-month follow up. Although in our group TNF-alpha had no relation to MIS score, our findings seem to be rather consistent with the abovementioned data. Based on our results, apart from age—which is unfortunately an unmodifiable factor, TNF-alpha is mostly linked to body composition parameters. As it is negatively associated with fat-free mass, we hypothesize that higher protein intake together with resistance training can be beneficial when it comes to diminishing its systemic effects and might also reduce mortality. Of course, higher levels of TNF-alpha in deceased in our study group might also be linked to their older age (mean: 59 vs. 69 in deceased). 

A very worthy, novel clinical trial by Catar et al. (Permeability Enhancement to Reduce Chronic Inflammation-II, NCT02084381) assessed the viability of high-flux vs. medium-cut off dialyzers in removing vascular endothelial growth factor (VEGF) during HD procedure. Not only did this study show the superiority of MCO dialyzers over high-flux, but also identified TNF-alpha as a catalyst for VEGF activation [50]. Thus, modifying dialysis-related parameters can also be a promising direction in repressing TNF-alpha signaling.

IL-1-alpha is also known as “alarmin”, as current data support its role in acute phase reaction and cell necrosis. Its elevated levels had previously been described in acute kidney injury with tubular necrosis or ANCA vasculitis [51]. Data on IL-1-alpha utility in chronic kidney disease is limited, but authors chose to assess the levels of this cytokine in this particular study as it was previously shown to promote anorexia in rats by suppressing appetite [52]. IL-6 administration also temporarily increased leptin levels in cancer patients in a dose-dependent manner [53]. Newer data show that higher levels of IL-1-alpha can also be seen in cancer cachexia [54]. Thus, we hypothesized that it could impact nutritional status in hemodialyzed patients, but in our study IL-1-alpha levels showed positive correlation only with TNF-alpha and IL-6 levels. None of the nutritional or erythropoiesis-related parameters we assessed had any link to IL-1-alpha. Nevertheless, it is worth noting that there are some data on the possible administration of bermekimab, a monoclonal antibody targeting IL-1alpha, in treating cancer cachexia by successfully improving lean mass [55]. The importance of IL-1a signaling in protein-energy wasting and MIS development in end-stage kidney disease still requires further studies.

Last but not least, we chose to assess IL-18 levels, as IL-18 is a part of NRLP3 inflammasome, whose role is currently gaining attention in the development of chronic kidney disease and its complications [56,57,58,59]. Bi et al. showed that higher IL-18 levels are linked to severity of PEW measured by albumin and pre-albumin levels in hemodialyzed [60]. In a study by Pourhassan et al., elevated levels of IL-18 were independently linked to decreased appetite in hospitalized patients [61]. Similar conclusions were drawn by Francesconi et al. in an animal model, where the administration of recombinant IL-18 inhibited food intake in mice for at least 6 h and had an anorexigenic effect by acting on neurons of the bed nucleus of the stria terminalis (BST) [62]. Chang stated that increase in IL-18 can be a predictor of cardiovascular events in hemodialyzed patients but emphasized that various chronic diseases can interfere with the result [56]. In some studies, elevated level of IL-18 was paired with increase in TNF-alpha in chronic kidney disease [59,63]. In our study group, IL-18 showed no significant correlation with any of the anthropometric or biochemical parameters. There are currently two substances available targeting IL-18 signaling: a monoclonal antibody GSK1070806 and a recombinant human endogenous IL-18 inhibitor, Tadekinig Alfa. Those were already investigated in delayed graft function, type 2 DM, Crohn’s disease, and Still’s disease [59]. Although our study did not elucidate the role of IL-18 in the development of malnutrition and erythropoietin resistance in ESRD, we are sure it might be an interesting area for our future, larger studies. 

## 5. Conclusions

Inflammation is an important contributor to the development of malnutrition and erythropoietin resistance in hemodialyzed individuals. Due to the multitude of inflammatory markers that can exacerbate MIS, multidirectional possible interventions need to be taken into account. Those can include: (1) modulation of adipokine- and cytokine-related signaling, (2) improving dialysis quality by choosing different membranes which would be more effective in removing uremic toxins, and (3) nutritional counselling [64]. Large clinical studies that target the inflammatory response in hemodialyzed patients, especially IL-6, TNF-alpha, and leptin, would be of great worth.

## 6. Limitations

This study relied on a relatively small sample size, as it was undertaken in a single dialysis center. Power analysis for such sample was not performed. Authors believe that repetitive blood sampling for cytokines and leptin levels during follow up, paired with body composition analysis, could add more valuable data concerning the dynamics of MIS and erythropoietin resistance development. Unfortunately, this could not be performed due to a lack of funds.

## Figures and Tables

**Table 1:** Group characteristics.

Overall Participants	n = 78
Male	n = 47 (60.3%)
Age	Median: 65; IQR = 21
Dialysis vintage [months]	Median: 28.5; IQR = 42
HD sessions per week	Median: 3; IQR = 0
ERI [IU/kg/g/dL/week]	Median: 4.9; IQR = 6.8
IL-6 [pg/mL]	Median: 3; IQR = 2.87
TNFα [pg/mL]	Median: 3.49; IQR = 2.17
IL-1α [pg/mL]	Median: 0.69; IQR = 0.55
IL-18 [pg/mL]	Median: 408.08; IQR = 209.03
Albumin [mg/mL]	Median: 41; IQR = 5
Transferrin [g/L]	Median: 1.7; IQR = 0.26
Ferritin [mcg/L]	Median: 475; IQR = 557
Hepcidin [ng/mL]	Median: 92.55; IQR = 108.8
Hemoglobin [mmol/L]	Mean: 6.72 (SD = 0.86)
Iron [mcg/dL]	Median: 65; IQR = 39
Leptin [ng/mL]	Median: 16.36; IQR = 51.77
Uric acid [mg/dL]	Mean: 6.24 (SD = 1.53)
Triglycerides [mg/dL]	Median: 149.5; IQR = 103
Total MIS score	Median: 5; IQR = 5
Kt/V	Mean: 1.14 (SD 0.23)
Patients’ nutrition by BMI [%]	underweight 2.6%; normal 26.9%; overweight 42.3%; obese 28.2%
Patients’ nutrition by SECA mBCA body composition chart [%]	increasing sarcopenic obesity: 23.2%; increasing obesity: 30.4%; increasing thinness: 17.4%; increasing muscle mass: 29%
eGFR [mL/min/1.73 m^2^]	Median: 7; IQR = 4

Abbreviations: IQR—interquartile range, BMI—body mass index, mBCA—medical body composition analyzer, ERI—erythropoietin resistance index, eGFR—estimated glomerular filtration rate, MIS—malnutrition inflammation scale.

**Table 2:** Association of leptin levels, nutritional parameters and biochemical data; n = 78.

	ρ (Rho)	*p*
leptin & body weight	0.497	<0.001
leptin & BMI	0.652	<0.001
leptin & BSA	0.358	0.001
leptin & MIS total score	−0.271	0.020
leptin & FFM	−0.518	<0.001
leptin & FM	0.518	<0.001
leptin & FMI	0.540	<0.001
leptin & muscle%	−0.425	<0.001
leptin & VAT	0.561	<0.001
leptin & TBW	−0.548	<0.001
leptin & ERI	−0.310	0.006
leptin & Fe	0.262	0.021
Leptin & TG	0.288	0.010
leptin & UA	0.301	0.007

Abbreviations: BMI—body mass index, BSA—body surface area, MIS—malnutrition inflammation scale, FFM—fat free mass, FM—fat mass, FMI—fat mass index, VAT—visceral adipose tissue, TBW—total body water, ERI—erythropoietin resistance index, Fe—iron, TG—triglycerides, UA—uric acid.

**Table 3:** General Linear Model (GLM) analysis of independent determinants of log-transformed leptin levels in terms of body composition and biochemical data; n = 78.

GLM Model (*p* = 0.0092)				
	*p*	Beta (β)	−95.00% CI Beta	+95.00% CI Beta
Sex (M vs. F)	0.0001	−0.378	−0.557	−0.198
Age	0.75	−0.025	−0.179	0.130
Uric acid	0.001	0.262	0.110	0.415
Log muscle%	0.025	−0.207	−0.386	−0.027
Log VAT	0.008	0.317	0.087	0.546
Log BMI	0.002	0.327	0.122	0.533

Abbreviations: VAT—visceral adipose tissue, BMI—body mass index.

**Table 4:** Comparison of leptin levels among groups with higher and lower erythropoietin resistance index (ERI). Median ERI in the whole study group was 4.89; n = 78.

		Mean	Median	Minimum	Maximum	Lower Quartile	Upper Quartile	IQR	SD
Leptin levels [ng/mL]	ERI lower than median	48.88	33.88	0.96	152.37	9.12	95.45	86.33	45.98
	ERI higher than median	23.47	7.36	0.50	134.68	2.70	22.50	19.80	33.73

Abbreviations: ERI—erythropoietin resistance index, IQR—interquartile range, SD—standard deviation.

**Table 5:** Association of TNFα levels, age, body composition and biochemical markers; n = 78.

	ρ (Rho)	*p*
TNFα & age	0.238	0.039
TNFα & FFM	−0.244	0.047
TNFα & FM	0.244	0.047
TNFα & FMI	0.246	0.045
TNFα & IL1α	0.233	0.043

Abbreviations: FFM—fat free mass, FM—fat mass, FMI—fat mass index.

**Table 6:** Association of IL-6 levels, body composition, biochemical parameters and dialysis-related data; n = 78.

	ρ (Rho)	*p*
IL-6 & ERI	0.229	0.046
IL-6 & phase angle	−0.261	0.033
IL-6 & HD sessions/week	0.282	0.014
IL-6 & dialysis vintage	0.229	0.047
IL-6 & IL-1α	0.359	0.002

Abbreviations: ERI—erythropoietin resistance index.

**Table 7:** General Linear Model (GLM) analysis of independent determinants of log-transformed ERI value in terms of body composition and biochemical data; n = 78.

GLM Model (*p* = 0.009)				
	*p*	Beta (β)	−95.00% CI Beta	+95.00% CI Beta
Sex (M vs. F)	0.735	0.037	−0.255	0.181
Age	0.736	0.038	−0.265	0.188
BMI	0.003	0.338	−0.559	−0.116
Log IL-6	0.029	0.248	0.026	0.470

Abbreviations: BMI—body mass index.

**Table 8:** Correlations of main cytokines in the study with ERI value.

	ρ	*p*-Value
ERI & TNFα	0.075	0.518
ERI & IL-6	0.229	0.046
ERI & IL-1α	0.128	0.271
ERI & IL-18	0.093	0.424
ERI & leptin	−0.310	0.006

**Table 9:** Correlations of main cytokines in the study with MIS score.

	ρ	*p*-Value
MIS & TNFα	0.212	0.063
MIS & IL-6	0.216	0.114
MIS & IL-1α	0.009	0.427
MIS & IL-18	0.090	0.641
MIS & leptin	−0.271	0.022

## Data Availability

Not applicable.
